# A missense variant rs2585405 in clock gene PER1 is associated with the increased risk of noise-induced hearing loss in a Chinese occupational population

**DOI:** 10.1186/s12920-021-01075-x

**Published:** 2021-09-08

**Authors:** Hao Chen, Xuexue Ding, Enmin Ding, Mengyao Chen, Huimin Wang, Guangzhi Yang, Baoli Zhu

**Affiliations:** 1 Center for Global Health, School of Public Health, Nanjing Medical University, Nanjing, 210000 Jiangsu China; 2 Institute of Occupational Disease Prevention, Jiangsu Province Center for Disease Prevention and Control, Nanjing, 21009 Jiangsu China; 3 Key Laboratory of Environmental Medicine Engineering of Ministry of Education, School of Public Health, Southeast University, Nanjing, 210003 Jiangsu China

**Keywords:** Cochlear clock genes, *PER1*, Polymorphism, Noise-induced hearing loss, Susceptibility

## Abstract

**Objective:**

To investigate the potential association of cochlear clock genes (*CRY1*, *CRY2*, *PER1*, and *PER2*), the *DNF* gene (brain-derived neurotrophic factor), and the *NTF3* gene (neurotrophin3) with susceptivity to noise-induced hearing loss (NIHL) among Chinese noise-exposed workers.

**Methods:**

A nested case–control study was performed with 2056 noise-exposed workers from a chemical fiber factory and an energy company who underwent occupational health examinations in 2019 as study subjects. Propensity score matching was conducted to screen cases and controls by matching sex, age, and the consumption of tobacco and alcohol. A total of 1269 participants were enrolled. Then, general information and noise exposure of the study subjects were obtained through a questionnaire survey and on-site noise detection. According to the results of audiological evaluations, the participants were divided into the case group (n = 432, high-frequency threshold shift > 25 dB) and the matched control group (n = 837, high-frequency threshold shift ≤ 25 dB) by propensity score matching. Genotyping for *PER1* rs2253820 and rs2585405; *PER2* rs56386336 and rs934945; *CRY1* rs1056560 and rs3809236; *CRY2* rs2292910 and rs6798; *BDNF* rs11030099, rs7124442 and rs6265; and *NTF3* rs1805149 was conducted using the TaqMan-PCR technique.

**Results:**

In the dominant model and the co-dominant model, the distribution of *PER1* rs2585405 genotypes between the case group and the control group was significantly different (*P* = 0.03, *P* = 0.01). The NIHL risk of the subjects with the GC genotype was 1.41 times the risk of those carrying the GG genotype (95% confidence interval (CI) of odds ratio (OR): 1.01–1.96), and the NIHL risk of the subjects with the CC genotype was 0.93 times the risk of those carrying the GG genotype (95%CI of OR: 0.71–1.21). After the noise exposure period and noise exposure intensities were stratified, in the co-dominant model, the adjusted OR values for noise intensities of ≤ 85 was 1.23 (95%CI: 0.99–1.53). In the dominant model, the adjusted OR values for noise exposure periods of ≤ 16 years and noise intensities of ≤ 85 were 1.88 (95%CI: 1.03–3.42) and 1.64 (95%CI: 1.12–2.38), respectively.

**Conclusion:**

The CC/CG genotype of rs2585405 in the *PER1* gene was identified as a potential risk factor for NIHL in Chinese noise-exposed workers, and interaction between rs2585405 and high temperature was found to be associated with NIHL risk.

**Supplementary Information:**

The online version contains supplementary material available at 10.1186/s12920-021-01075-x.

## Background

Noise-induced hearing loss (NIHL), which ranks the second among the forms of sensorineural hearing loss worldwide [1], is an occupational health hazard worldwide. NIHL is widely acknowledged as a complicated disease arising from the synergistic effect of inherited factors and environmental factors [2]. The World Health Organization and The National Institute of Occupational Safety and Health have listed NIHL as a research focus. The pathogenesis of NIHL is not completely revealed at the moment. Possible hypotheses for the pathogenesis include the overloaded calcium channels, which stimulates cell apoptosis [3–5]; the production of free radicals or reactive oxygen species [6–8]; and glutamate excitotoxicity, activating signaling pathways that lead to cell death [9]. Variations of NIHL vulnerability exhibited in individuals had been indicated by previous human and animal research [10, 11].

The production of circadian rhythm is a process involving cells. The transcription and expression of *PERs* and *CRYs* are irritated by core clock genes *CLOCK* and *BMAL1*, the products of *PERs* and *CRYs* combined with *CLOCK* and *BMAL1* to inhibit its own transcription and expression [12, 13]. When the products reach a certain level, inhibition stops, and *CLOCK* and *BMAL1* start a new transcription cycle [14, 15]. Previous studies reported that auditory function could be affected by circadian rhythm [16] and that self-sustaining circadian rhythm exists in the cochlea [17, 18]. Animal research indicated that the expression of core clock genes could be affected by noise, resulting in the disturbance of circadian rhythm and impairing auditory function [16–18]. Furthermore, some studies revealed that both *PER1* and *PER2* were expressed in the cochlea and inferior colliculus [17, 18]. *BDNF* and *NTF3* belong to the nerve growth factor (NGF) protein family [19], with similar structures [20]. Previous research showed that *BDNF* and *NTF-3* played important roles in the growth and development of spiral ganglion neurons (SGN). During the development of the cochlea, neurotrophic factors regulate the differentiation and survival of neurons [21–24].

Clock genes also play an important role in other human diseases. Loss of the clock gene *Per1* promoted oral squamous cell carcinoma progression [25]. The loss of function of *Per1* or *Per2* led to premature ovarian insufficiency [26]. *CRY1* is a tumor-specific regulator of DNA repair related to poor prognosis in patients with prostate cancer [27]. Dysfunctional expression of *CRY2* is associated with the susceptibility to depression [28]. A previous study revealed that the association of sleep disturbance and disrupted circadian rhythm led to sudden sensorineural hearing loss (SSNHL), with a lower expression of *CRY1* and *CRY2* [28]. As far as we know, there has been no research on the correlation between clock genes and NIHL susceptibility.

The purpose of this study was to explore the genetic association between SNPs (single nucleotide polymorphisms) of clock and neurotrophic factor genes and the susceptibility of the Chinese Han population to NIHL, providing clues for research on genetic susceptibility to NIHL.

## Material and methods

### Subjects

The source of subjects for this study was a cohort study that started in 2012. Employees from a textile factory and an energy company in eastward China who received physical health examinations once a year carried by the Jiangsu Provincial Center for Disease Prevention and Control were enlisted for our study in 2019. The inclusion criteria were (1) Chinese Han workers (2) with more than 3 years of noise exposure (hereinafter referred to as "noise exposure"); (3) exposure to noise with other harmful factors (e.g., high temperature, vibration, organic solvents, and carbon monoxide) that may affect the onset of NIHL that was below the occupational exposure limit (GBZ 2.1-2007; GBZ 2.2-2007); and (4) with workplace monitoring data and integral health surveillance data. The exclusion criteria were (1) a history of taking ototoxic drugs and family history of hereditary deafness and other head trauma, (2) people with ear diseases such as ear canal deformity, tympanic membrane perforation. The study was supported by the Ethical Committee of the Jiangsu Provincial Center for Disease Prevention and Control and informed consent was obtained from the research subjects.

### Research design

A nested case–control design was performed in this study. The participants in this study were part of a cohort that started in 2012, which included employees exposed to noise in a textile factory and an energy company in eastern China by the Jiangsu Provincial Center for Disease Prevention and Control who had annual follow-up examinations. Our research participants were the 2019 cohort of the study begun in 2012.

### Questionnaire investigation

This study used the *noise-induced hearing loss questionnaire* designed by the research group*.* The content of the questionnaire included (1) informed consent; (2) basic information (marriage, sex, age, and education); (3) tobacco and alcohol consumption habits; (4) current and past medical history (e.g., hypertension, diabetes, hyperlipidemia, and ear diseases); and (5) occupational noise exposure history (work years, noise exposure years, and protective measures).

### Pure-tone audiometry (PTA) testing and environmental noise measurement

The pure-tone audiometry was examined applying A Madsen Voyager 522 audiometer (Madsen, Taastrup, Denmark). The binaural hearing test was conducted for all participants after staying away from noise for at least 48 h to eliminate the effect of temporary hearing threshold shift on the results. Then, each subject was examined at 6 frequencies of 500, 1000, 2000, 3000, 4000, and 6000 Hz, respectively in an soundproof room. The formular for calculating binaural high frequency average hearing threshold is as follows.

$$\text{Binaural high frequency average hearing threshold}=\frac{left\left({HL}_{3KHZ}+{HL}_{4KHZ}+{HL}_{6KHZ}\right)+righ\left({HL}_{3KHZ}+{HL}_{4KHZ}+{HL}_{6KHZ}\right)}{6},$$

*left* is left ear; *righ* is right ear; *HL* is missing value of 6 frequencies of 500, 1000, 2000, 3000, 4000, and 6000 Hz, dB.

Noise exposure level was evaluated in each working point using noise dosimeters (Noise-Pro; Quest, Oconomowoc, WI, USA) by the direction of the Chinese National Criteria for Noise at the Workplace. Cumulative noise exposure (CNE) was applied to determine noise exposure level of individuals in this study. The calculation of CNE is based on the 8-h equivalent continuous sound level (A) in the operating points, and the formular for calculating CNE is as follows.

$$\mathrm{CNE}=10\mathrm{log}\left[\frac{1}{{T}_{ref}}\sum_{i=1}^{n}\left({T}_{i}\times {10}^{{L}_{Aeq,8h/10}}\right)\right],$$

*T*_*ref*_ refers to 1; *n* is equivalent to overall number of different work position exposed to noise for the workers; $i$ is equal to the number of different posts; *T* refers to consumption of the time at various locations; *L *_*Aeq,8 h*_ is equal to sequential sound level of 8 h for different types of work.

### Definition of NIHL and control subjects and propensity score matching

Hearing abnormity and hearing normality were determined on the basis of the Chinese diagnostic criteria applying to occupational noise-induced deafness (GBZ 49–2007). In this study, hearing loss was identified using binaural high-frequency hearing thresholds outside of 25 dB(A). Correspondingly, hearing normality was identified by a binaural high-frequency hearing threshold below of 25 dB(A). The hearing threshold values were obtained by PTA testing. All participants were divided into NIHL and normal hearing groups. The NIHL individuals refer to noise-exposed individuals with hearing abnormity and the control individuals were determined as noise-exposed individuals with hearing normality. The NIHL patients were selected first, and the controls were matched to the patients according to sex, age, and noise exposure using propensity score matching at a ratio of 1:2. Eventually, 432 NIHL patients and 837 controls were selected from all participants.

### Extraction of DNA and selection of SNPs and genotyping

Peripheral blood (2 ml) of the subjects was drawn into ethylene diamine tetraacetic acid (EDTA) tubes, centrifuging 3000 r/min for 5 min to extract DNA for genotyping. The isolation of DNA from the peripheral blood was conducted by the QIA cube HT and the QIA amp 96 DNA QIA cube HT Kit (Qiagen, Dusseldorf, Germany). Subsequent to the isolation, the DNA samples were stored at − 20 °C until use.

The selection of the SNPs in the target genes was from the National Center for Biotechnology Information (NCBI) database (http://www.ncbi.nlm.nih.gov/) and previous literature using the standard of a minimum allele frequency (MAF) of > 0.05 and present in a Chinese Han population. In accordance with the standard, 12 SNPs met the criteria (rs2253820, rs2585405, rs56386336, rs934945, rs1056560, rs3809236, rs2292910, rs6798, rs11030099, rs7124442, rs6265rs, and 1805149). All these SNPs are located in the functional areas of the chromosomes.

ABI TaqMan SNP genotyping assays (Applied Biosystems, Foster City, CA, USA) and genotyping probes and primers that designed commercially were adopted to confirm the genotypes of the selected SNPs. The designed commercial products were mixed with TaqMan Universal PCR Master Mix (Roche, Branchburg, NJ, USA) respectively following the attached guideline. After thawing, the extracted DNA were added into mixture. A QuantStudio™ 7 Flex System (Applied Biosystems) was used to perform the genotyping. The results were analyzed using QuantStudio™ 7 Flex System Sequence Detection software version 1.2.3 (Applied Biosystems).

### Statistical analyses

Statistical analysis was conducted with the assistance of SAS 9.4 software. The continuous variables and categorical variables were described as mean ± SD and percentages respectively. The differences in age, years of exposure to noise, and the intensity of noise exposure between the NIHL and control groups were evaluated using Student’s t-tests or paired t-tests. The differences in sex, tobacco and alcohol consumption, and genotype distribution among the two groups were compared using the χ^2^ test. Hardy–Weinberg equilibrium in the target SNPs of the participants were calculated by goodness-of-fit χ^2^ tests. The odds ratios (ORs) with 95% confidence intervals (95% CI) of the subjects who were different genotypes were defined using conditional logistic regression models adjusted for age, sex, smoking, and drinking. The interaction analysis between rs2585405 and high temperature was performed using MDR 3.0.2 software. Propensity score matching was conducted using R 4.0.5 software. Sensitivity analysis was performed using SAS 9.4 software. The Bonferroni method was used to correct all *P*-values, and statistical significance was determined as *P* < 0.05.

## Results

### Basic information of study subjects and Hardy–Weinberg tests of selected SNPs

General information (sex and age), life habit characteristics (consumption of tobacco and alcohol, noise exposure, and noise-intensity), and high-frequency hearing threshold shifts are shown in Table 1. No significant difference was found in the general information and lifestyle features between the two groups. However, there was a significant difference in high-frequency hearing threshold shifts between the NIHL and control groups (*P* < 0.001). General information on the selected SNPs and the results of the Hardy-Weinberg test are shown in Table 2. All selected SNPs had minor allele frequencies of ≥ 5% and were in Hardy-Weinberg equilibrium (HWE) (*P* > 0.05), indicating that the sample selected in this study was representative of the group and that the gene frequency of the research objects could represent the gene distribution of the population.

**Table 1:** Demographic characteristics of study subjects

Variables	Cases (n = 432)		Controls (n = 837)		*P*
	n	%	n	%	
*Age (years)*					0.09^a^
Mean ± SD	47.85 ± 5.48		47.43 ± 5.38		0.18^b^
≤ 35	13	1.0	26	2.1	
35–45	89	7.0	219	17.3	
> 45	330	26.0	592	46.7	
*Sex*					0.40^a^
Male	392	30.9	771	60.8	
Female	40	3.2	66	5.2	
*Tobacco use*					0.93^a^
Now	194	15.3	372	29.3	
Ever	170	13.4	338	26.6	
Never	68	5.4	127	10.0	
*Alcohol consumption*					0.79^a^
Now	192	15.1	356	28.1	
Ever	33	2.6	63	5.0	
Never	207	16.3	418	32.9	
*Duration of noise exposed work (years)*					0.75^a^
Mean ± SD	23.72 ± 10.58		23.68 ± 10.55		0.95^b^
≤ 16	84	6.6	169	13.3	
> 16	348	27.4	668	52.6	
*Noise exposure levels (dB)*					0.17^a^
≤ 85	265	20.9	474	37.4	
85–92	126	9.9	288	22.7	
> 92	41	3.2	75	5.9	
*High frequency hearing thresholds (dB)*					** < 0.001** ^**a**^
Mean ± SD	0.64 ± 0.48			0	** < 0.001** ^**b**^
≤ 26	155	12.2	837	66.0	
> 26	277	21.4	0	0.0	

^a^Students’ t-test

^b^Two-sided χ^2^ test

**Table 2:** General information of selected SNPs and Hardy–Weinberg test

Gene	SNP	Alleles	Chromosome	Functional Consequence	MAF database^a^	*P* for HWE^b^
*PER1*	rs2253820	A/G	17:8144851	splice region variant	0.44	0.954
	rs2585405	C/G	17:8143454	missense variant	0.23	0.441
*PER2*	rs56386336	A/G	2:238245307	3'prime UTR variant	0.09	1.000
	rs934945	A/G	2:238246412	missense variant	0.17	0.026
*CRY1*	rs1056560	G/T	12:106991832	3'prime UTR variant	0.47	0.402
	rs3809236	C/T	12:107093269	5'prime UTR variant	0.20	0.892
*CRY2*	rs2292910	C/A	11:45882062	3'prime UTR variant	0.42	0.994
	rs6798	C/T	11:45882926	3'prime UTR variant	0.24	0.892
*BDNF*	rs11030099	A/C	11:27656036	3'prime UTR variant	0.23	0.205
	rs7124442	C/T	11:27655494	3'prime UTR variant	0.33	0.654
	rs6265	A/G	11:27658369	missense variant	0.20	0.264
*NTF3*	rs1805149	A/G	12:5494441	missense variant	0.12	0.592

^a^Data from NCBI dbSNP

^b^*P* value of Hardy–Weinberg test

### Multivariate analysis of selected SNPs with the risk of NIHL

Twelve SNPs were determined in 1269 workers exposed to noise (432 NIHL patients and 837 controls). The results of the genotypes and allele distributions of the twelve SNPs are shown in Table 3. Analysis of the selected SNPs in 4 gene models (codominant, dominant, recessive, and allelic models) showed statistically significant differences in the genotype frequencies of gene *PER1* rs2585405 between the cases and controls (*P* = 0.032 and *P* = 0.01, respectively) in the codominant and dominant models. In the codominant model, the GC and CC genotypes were risk factors for the onset of NIHL, and individuals with GC or CC had an increased risk of NIHL (OR = 1.41, 95%CI: 1.01–1.96, OR = 0.93, 95%CI: 0.71–1.21, respectively) by logistic regression analysis after adjusting for age, sex, and alcohol and tobacco consumption habits. In the dominant model, logistic regression analysis after adjusting for age, sex, and alcohol and tobacco consumption habits demonstrated that individuals with the GC/CC genotype had a significantly increased risk for NIHL (OR = 1.47, 95%CI: 1.10–1.97). Thus, our data revealed that the gene *PER1* rs2585405 may have a significant association with increased NIHL susceptibility.

**Table 3:** Distribution of selected SNPs and the association with NIHL

Gene	Genetic models	Genotypes	Cases		Controls		*P*^a^	FDR	Adjusted OR (95%CI)^b^
			n = 432	%	n = 837	%			
*PER1*	rs2585405								
	Codominant	GG	98	22.7	140	16.7	**0.03**	0.36	1.00 (Ref.)
		GC	198	45.8	425	50.8			**1.41 (1.01–1.96)**
		CC	136	31.5	272	32.5			**0.93 (0.71–1.21)**
	Dominant	GG	98	22.7	140	16.7	**0.01**	0.12	1.00 (Ref.)
		GC/CC	334	77.3	697	83.3			**1.47 (1.10–1.97)**
	Recessive	GG/GC	296	68.5	565	67.5	0.71	0.97	1.00 (Ref.)
		CC	136	31.5	272	32.5			1.05 (0.82–1.34)
	Alleles	G	194	31.0	705	55.6	0.09	0.88	1.00 (Ref.)
		C	470	37.0	969	76.4			1.15 (0.98–1.36)
	rs2253820								
	Codominant	GG	44	10.2	96	11.5	0.45	0.98	1.00 (Ref.)
		AG	196	45.4	399	47.7			0.82 (0.55–1.22)
		AA	192	44.4	342	40.9			0.87 (0.68–1.12)
	Dominant	GG	44	10.2	96	11.5	0.49	0.95	1.00 (Ref.)
		AA/AG	388	89.8	741	88.5			0.88 (0.60–1.28)
	Recessive	AG/GG	240	55.6	495	59.1	0.22	0.97	1.00 (Ref.)
		AA	192	44.4	342	40.9			0.86 (0.68–1.09)
	Alleles	G	284	22.4	591	46.6	0.22	0.88	1.00 (Ref.)
		A	580	45.7	1083	85.3			0.89 (0.75–1.07)
*PER2*	rs56386336								
	Codominant	GG	332	76.9	661	79.0	0.50	0.98	1.00 (Ref.)
		AG	96	22.2	165	19.7			1.26 (0.40–4.02)
		AA	4	0.9	11	1.3			1.47 (0.45–4.78)
	Dominant	GG	332	76.9	661	79.0	0.39	0.95	1.00 (Ref.)
		AG/GG	100	23.1	176	21.0			0.88 (0.66–1.16)
	Recessive	GG/AG	428	99.1	826	98.7	0.54	0.97	1.00 (Ref.)
		AA	4	0.9	11	1.3			1.31 (0.41–4.15)
	Alleles	G	760	59.9	1487	117.1	0.52	0.90	1.00 (Ref.)
		A	104	8.2	187	14.7			1.10 (0.85–1.43)
	rs934945								
	Codominant	GG	242	56.0	458	54.7	0.75	0.99	1.00 (Ref.)
		AG	157	36.3	305	36.4			1.19 (0.77–1.85)
		AA	33	7.6	74	8.8			1.16 (0.73–1.82)
	Dominant	GG	242	56.0	458	54.7	0.66	0.95	1.00 (Ref.)
		AG/AA	190	44.0	379	45.3			1.06 (0.84–1.34)
	Recessive	AG/GG	399	92.4	763	91.2	0.47	0.97	1.00 (Ref.)
		AA	33	7.6	74	8.8			1.18 (0.77–1.81)
	Alleles	G	641	50.5	1221	96.2	0.50	0.90	1.00 (Ref.)
		A	223	17.6	453	35.7			0.94 (0.78–1.13)
*CRY1*	rs1056560								
	Codominant	GG	25	5.8	54	6.5	0.37	0.98	1.00 (Ref.)
		GT	143	33.1	306	36.6			0.83 (0.51–1.37)
		TT	264	61.1	477	57.0			0.84 (0.66–1.08)
	Dominant	GG	25	5.8	54	6.5	0.64	0.95	1.00 (Ref.)
		GT/TT	407	94.2	783	93.5			0.89 (0.54–1.45)
	Recessive	GT/GG	168	38.9	360	43.0	0.16	0.97	1.00 (Ref.)
		TT	264	61.1	477	57.0			0.84 (0.66–1.07)
	Alleles	G	193	15.2	414	326	0.18	0.88	1.00 (Ref.)
		T	671	52.9	1260	99.3			0.88 (0.72–1.06)
	rs3809236								
	Codominant	CC	276	63.9	514	61.4	0.59	0.98	1.00 (Ref.)
		TC	139	32.2	282	33.7			1.32 (0.73–2.37)
		TT	17	3.9	41	4.9			1.21 (0.66–2.22)
	Dominant	CC	276	63.9	514	61.4	0.39	0.95	1.00 (Ref.)
		TC/TT	156	36.1	323	38.6			1.11 (0.87–1.42)
	Recessive	TC/CC	415	96.1	796	95.1	0.44	0.97	1.00 (Ref.)
		TT	17	3.9	41	4.9			1.28 (0.72–2.29)
	Alleles	C	691	54.5	1310	103.2	0.31	0.90	1.00 (Ref.)
		T	173	13.6	364	28.7			0.90 (0.73–1.10)
*CRY2*	rs2292910								
	Codominant	CC	36	8.3	75	9.0	0.90	0.99	1.00 (Ref.)
		AC	180	41.7	340	40.6			0.93 (0.61–1.43)
		AA	216	50.0	422	50.4			1.04 (0.81–1.32)
	Dominant	CC	36	8.3	75	9.0	0.71	0.95	1.00 (Ref.)
		AC/AA	396	91.7	762	91.0			0.92 (0.61–1.39)
	Recessive	AC/CC	216	50.0	415	49.6	0.89	0.97	1.00 (Ref.)
		AA	216	50.0	422	50.4			1.02 (0.81–1.28)
	Alleles	C	252	19.9	490	38.6	0.96	0,99	1.00 (Ref.)
		A	612	48.2	1184	93.3			0.99 (0.83–1.19)
	rs6798								
	Codominant	CC	82	19.0	173	20.7	0.60	0.98	1.00 (Ref.)
		TC	218	50.5	398	47.6			0.96 (0.69–1.35)
		TT	132	30.6	266	31.8			1.10 (0.84–1.44)
	Dominant	CC	82	19.0	173	20.7	0.48	0.95	1.00 (Ref.)
		TC/TT	350	81.0	664	79.3			0.91 (0.68–1.22)
	Recessive	TC/CC	300	69.4	571	68.2	0.66	0.97	1.00 (Ref.)
		TT	132	30.6	266	31.8			1.06 (0.82–1.37)
	Alleles	C	382	30.1	744	58.6	0.91	0.99	1.00 (Ref.)
		T	482	38.0	930	73.3			0.99 (0.84–1.17)
*BDNF*	rs11030099								
	Codominant	CC	139	32.2	250	29.9	0.65	0.98	1.00 (Ref.)
		AC	195	45.1	398	47.6			1.08 (0.78–1.48)
		AA	98	22.7	189	22.6			0.95 (0.70–1.28)
	Dominant	CC	139	32.2	250	29.9	0.40	0.95	1.00 (Ref.)
		AC/AA	293	67.8	587	70.1			1.11 (0.87–1.43)
	Recessive	AC/CC	334	77.3	648	77.4	0.97	0.97	1.00 (Ref.)
		AA	98	22.7	189	22.6			1.00 (0.76–1.32)
	Alleles	C	473	37.3	898	70.8	0.60	0.90	1.00 (Ref.)
		A	391	30.8	776	61.2			0.96 (0.82–1.12)
	rs7124442								
	Codominant	CC	1	0.2	2	0.2	0.95	0.99	1.00 (Ref.)
		TC	58	13.4	107	12.8			0.89 (0.08–9.93)
		TT	373	86.3	728	87.0			1.06 (0.75–1.50)
	Dominant	CC	1	0.2	2	0.2	1.00	1.00	1.00 (Ref.)
		TC/TT	431	99.8	835	99.8			0.88 (0.08–9.85)
	Recessive	TC/CC	59	13.7	109	13.0	0.75	0.97	1.00 (Ref.)
		TT	373	86.3	728	87.0			1.06 (0.75–1.49)
	Alleles	C	60	4.7	111	8.7	0.77	0.99	1.00 (Ref.)
		T	804	63.4	1563	123.2			1.05 (0.75–1.46)
	rs6265								
	Codominant	GG	125	28.9	240	28.7	0.99	0.99	1.00 (Ref.)
		AG	204	47.2	399	47.7			1.00 (0.73–1.38)
		AA	103	23.8	198	23.7			0.99 (0.74–1.33)
	Dominant	GG	125	28.9	240	28.7	0.92	1.00	1.00 (Ref.)
		AA/AG	307	71.1	597	71.3			1.01 (0.78–1.30)
	Recessive	AG/GG	329	76.2	639	76.3	0.94	0.97	1.00 (Ref.)
		AA	103	23.8	198	23.7			0.99 (0.76–1.31)
	Alleles	G	454	35.8	879	69.3	0.99	0.99	1.00 (Ref.)
		A	410	32.3	795	62.6			1.00 (0.85–1.17)
*NTF3*	rs1805149								
	Codominant	AA	97	22.5	188	22.5	0.54	0.98	1.00 (Ref.)
		AG	67	15.5	111	13.3			1.02 (0.76–1.36)
		GG	268	62.0	538	64.3			1.20 (0.86–1.69)
	Dominant	AA	97	22.5	188	22.5	1.00	1.00	1.00 (Ref.)
		AG/GG	335	77.5	649	77.5			0.98 (0.74–1.30)
	Recessive	AG/AA	164	38.0	299	35.7	0.43	0.97	1.00 (Ref.)
		GG	268	62.0	538	64.3			0.50 (1.09–0.85)
	Alleles	A	261	20.6	487	38.4	0.56	0.90	1.00 (Ref.)
		G	603	47.5	1187	93.6			1.02 (0.89–1.18)

^a^Two-sided χ^2^ test

^b^Adjusted for age, sex, alcohol, and tobacco use in a logistic regression model

### Stratified analysis of rs2585405 polymorphism and NIHL risk

The results of stratified analysis of rs2585405 polymorphism and NIHL risk are shown in Table 4. In the group with noise exposure levels of ≤ 85 dB, the distribution of rs2585405 genotypes was significantly different between the cases and controls (*P* = 0.03), and individuals with GC + CC genotypes had an increased NIHL risk (OR = 1.23, 95%CI: 0.99–1.53). In the dominant model, in the group with noise exposures of ≤ 16 and noise intensities of ≤ 85 dB, statistically significant differences were found in the rs258540 genotype distributions between the cases and controls in (*P* = 0.04 and *P* = 0.01, respectively), and the NIHL risk of individuals with GC/CC genotypes between the cases and controls increased from 1.47 times before stratification (OR = 1.47, 95%CI: 1.10–1.97) to 1.88 times (OR = 1.88, 95%CI: 1.03–3.42) and 1.64 times (OR = 1.64, 95%CI: 1.12–2.38).

**Table 4:** Stratified analyses of rs2585405 polymorphism and NIHL risk

Group	Genotype	noise exposed (years)		Expose level with noise (dB)		
		≤ 16	> 16	≤ 85	85–92	> 92
Case	GG	27	71	63	26	9
	GC	33	165	124	59	15
	CC	24	112	78	41	17
Control	GG	34	106	76	55	9
	GC	80	345	248	139	38
	CC	55	217	150	94	28
*P*^a^		0.11	0.17	**0.03**	0.93	0.23
Adjusted OR		1.33	1.11	**1.23**	1.02	1.07
(95%CI)^b^		(0.93–1.90)	(0.92–1.34)	**(0.99–1.53)**	(0.76–1.37)	(0.61–1.87)
Case	GG	27	71	63	26	9
	GC/CC	57	277	202	100	32
Control	GG	34	106	76	55	9
	GC/CC	135	562	398	233	66
*P*^a^		**0.04**	0.07	**0.01**	0.72	0.16
Adjusted OR		**1.88**	1.37	**1.64**	1.10	1.92
(95%CI)^b^		**(1.03–3.42)**	(0.98–1.91)	**(1.12–2.38)**	(0.65–1.85)	(0.67–5.52)

^a^Two-sided χ^2^ test

^b^Adjusted for age, sex, alcohol and tobacco use in a logistic regression model

### Multifactor dimensionality reduction analysis of the interaction between rs2585405 and high temperature

**Fig. 1:**
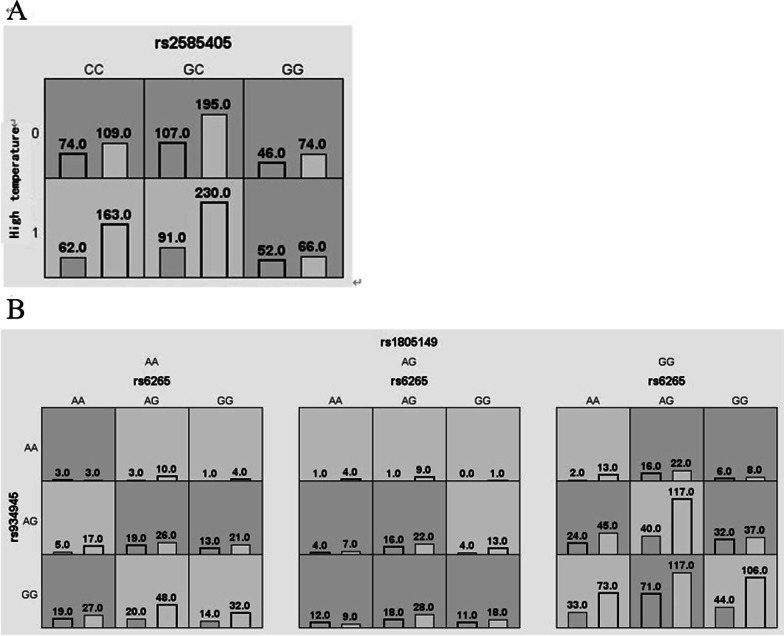
Graph model of the interaction between rs2585405 and high temperature (**A** rs2585405*High temperature model, **B** rs6265*rs934945*rs1805149 model). Dark gray and light gray boxes presented the high- and low-risk factor combinations, respectively. Left bars within each box represented case while the right bars represented control. The heights of the bars are proportional to the sum of samples in each group

The results of the multifactor dimensionality (MDR) analysis of the interaction between rs2585405 and high temperature are shown in Table 5 and Fig. 1. The results indicated that the rs2585405*high temperature and rs6265*rs934945*rs1805149 models were associated with NIHL risk (OR = 1.61 and 1.80, respectively, *P* < 0.001), which indicated that high temperature and rs2585405 were interactive risk factors for NIHL.

**Table 5:** Multifactor dimensionality reduction analysis of the interaction between rs2585405 and high temperature

Models	Training balanced accuracy	Testing balanced accuracy	Cross svalidation consistency	*P*	OR (95%CI)
rs2585405*High temperature	0.559	0.533	7/10	< 0.0001	1.61 (1.27–2.05)
rs6265*rs934945*rs1805149	0.579	0.501	3/10	< 0.0001	1.80 (1.42–2.28)

### Sensitivity analysis

The results of the sensitivity analysis were consistent with our analysis. The sensitivity analysis results are attached to Additional files 1–4.

## Discussion

SNPs are considered to be universal genetic variations in the human genome, and there are as many as 15 million SNPs among all humans [29]. SNPs are unevenly distributed in the genome. The frequency of SNPs in non-coding regions is higher than that in the gene coding regions. The present methods of detecting SNPs mainly include denaturing gradient gel electrophoresis, single-strand conformational polymorphism analysis, cleaved amplified polymorphic sequence assays, denaturing gradient gel electrophoresis, and allele-specific PCR (Taq-Man SNP genotype-PCR).

In this study, a large sample of Han nationality noise workers was preliminarily analyzed for the association between a total of twelve SNPs among clock and nerve growth factor genes and susceptibility to NIHL. The results showed that the distribution of the *PER1* gene rs2585405 genotypes between the case and control groups was statistical different in both the co-dominant model and the dominant model. The risk of NIHL in individuals with the GC/CC genotype was 0.47 times that of people with the GG genotype (adjusted OR = 0.47, 95%CI: 1.10–1.97), indicating that the GC/CC genotype at rs2585405 may be a risk factor for the onset of NIHL.

Sensory information is transmitted from the cochlea to the brainstem by spiral ganglion neurons that are susceptible to noise, senile, and underlying genetic diseases [30]. Evidence has shown that neurotrophic factors played an important role in the treatment of hearing loss. Studies have shown that *BDNF* and *NTF-3* were essential for establishing synapses and maintaining hearing function throughout life [31, 32]. Unfortunately, the SNPs screened in *BDNF* and *NTF-3* in this study did not identify any sites related to NIHL susceptibility.

In the research of Siwei Chen et al., the De novo missense variants in Autism Spectrum Disorder (ASD) patients could impact the risk of ASD by destroying interaction of protein–protein, which is a universal phenomenon in various diseases [33, 34].Dual-oxidase maturation factor 2 (DUOXA2) is a component of thyroid hydrogen peroxidase (H2O2) generator, which is essential to hormone synthesis. Congenital hypothyroidism happened with the genetic defect of DUOXA2 causing the damage of H2O2 production system. A novel DUOXA2 missense mutation (I26M) causes goiter that affected H2O2 production but did not alter protein expression levels [35, 36].

Missense mutation refers to a change in a codon that encodes a certain amino acid to a codon that encodes for another amino acid after a base substitution, which changes the type and sequence of amino acids in the polypeptide chain. As a result of missense mutations, the polypeptide chain usually loses its original function. Many protein abnormalities are caused by missense mutations [37]. Studies had reported that a missense variant in *PER2* was related to delayed sleep–wake phase disorder in a Japanese population [38]. A previous study indicated that a missense variant of *PER1* rs2585405 was associated with the susceptibility to prostate cancer [39]. However, there have been no studies on the correlation between clock genes and NIHL susceptibility. Therefore, in future research, the relevant mechanism of this locus can be explored and more clues on the influence of clock genes on NIHL can be revealed.

The circadian rhythm process depends upon a transcriptional feedback loop initiated by the heterodimeric factor *CLOCK: BMAL1* [40]. *CLOCK: BMAL1* activates the transcription and expression of core clock genes *Period* (*Per1*, *Per2*, *Per3*) and *Cryptochrome* (*Cry1* and *Cry2*). With sufficient expression products, *PERs* and *CRYs* form a complex to inhibit the transcription process mediated by *CLOCK: BMAL1* [41–44]*.* In animal research, the expression of clock genes was inhibited when the circadian rhythm in mice cochlea was disturbed. In addition, mice with normal circadian rhythms in a noisy environment showed higher threshold changes [45]. It had been reported that patients with sudden sensorineural hearing loss showed changes in biological clock genes [46]. These clues suggest that in the prevention strategies of NIHL patients, workers who are exposed to night shift noise should pay more attention to changes in hearing levels.

The research about the interaction between environmental factors and biological factors is essential to reveal response of specific SNPs to environmental factors. As an important biological factor in post-transcriptional processes, mRNA regulates various biological processes [47–50]. N4-Acetylcytidine was found being existing broadly on mRNA, which improved stability of mRNA and efficiency of protein translation [51–53]. Some studies have shown that mRNA plays an important role in the occurrence and development of hearing loss [47]. It had been suggested that the networks of microRNA–messenger RNA interactions existed in age-related hearing loss on the basis of researches in aging mice [54, 55].

As far as we know, this study is the first to investigate the correlation between clock gene polymorphisms and NIHL susceptibility, but the study had some shortcomings. First, although the number of samples in our study was comparatively large compared to previous studies, the power of the statistical test was not adequate to confirm the small biological effects of a single SNP possibility. Therefore, larger sample sizes and cohort studies are needed to confirm the impact of clock gene polymorphisms on NIHL risk in the future. Secondly, the subjects in this case–control study were limited to a Chinese population, the trans-ethnic meta-analysis and subgroup meta-analysis according to ethnicity, sex, gene-dosage, and age, based on the selected SNPs was not available to conducted for the limited genotype data [56–60]. Therefore, our results may be more applicable to the Chinese Han population, but the application to other populations may be limited. Thirdly, although the statistical significance between rs2585405 and NIHL susceptibility had been found in our study, it is still not able to determine the disease by the variant. So, the machine-learning model is relatively essential to predict disease risk based the significant SNP in the future research [61, 62]. Fourthly, the causality of the genetic variant in the development of noise-induced hearing loss was not revealed clearly in our research. So, the genotype data can be integrated with eQTL from GTEX or pQTLs to explore whether the polymorphisms in these genes are causally triggering the development of noise-induced hearing loss through mediating the expression of these genes in specific tissues in further study [63–66].

## Conclusions

In conclusion, the G allele of the *PER1* gene rs2585405 was associated with NIHL risk, and the site interacts with the factor of high temperature. Therefore, our results indicated that *PER1* rs2585405 may play a key role in NIHL and may serve as a biomarker for workers exposed to noise. In addition, this research provides a new research direction that an imbalance in circadian rhythm can impair auditory function.

## Supplementary Information

**Additional file 1** Table 6 Sensitivity analysis results of deleting age. The results of sensitivity analysis adjusted for sex, alcohol and tobacco use in a logistic regression model.

**Additional file 2** Table 7 Sensitivity analysis results of deleting tobacco. The results of sensitivity analysis adjusted for age, sex and alcohol use in a logistic regression model.

**Additional file 3** Table 8 The results of sensitivity analysis in stratified analyses deleting age. The results of sensitivity analysis in stratified analyses Adjusted for sex, alcohol and tobacco use in a logistic regression model.

**Additional file 4** Table 9 The results of sensitivity analysis in stratified analyses deleting tobacco. The results of sensitivity analysis in stratified analyses adjusted for age, sex and alcohol use in a logistic regression model.

## Data Availability

https://github.com/EnminDing/PER1.

## References

[1] Carlsson PI, Van Laer L, Borg E, Bondeson ML, Thys M, Fransen E (2005). The influence of genetic variation in oxidative stress genes on human noise susceptibility. Hear Res.

[2] Sliwinska-Kowalska M, Pawelczyk M (2013). Contribution of genetic factors to noise-induced hearing loss: a human studies review. Mutat Res.

[3] Fridberger A, Flock A, Ulfendahl M, Flock B (1998). Acoustic overstimulation increases outer hair cell Ca^2+^ concentrations and causes dynamic contractions of the hearing organ. Proc Natl Acad Sci USA.

[4] Wang X, Zhu Y, Long H, Pan S, Xiong H, Fang Q (2018). Mitochondrial calcium transporters mediate sensitivity to noise-induced losses of hair cells and cochlear synapses. Front Mol Neurosci.

[5] Orrenius S, Zhivotovsky B, Nicotera P (2003). Regulation of cell death: the calcium-apoptosis link. Nat Rev Mol Cell Biol.

[6] Yamane H, Nakai Y, Takayama M, Iguchi H, Nakagawa T, Kojima A (1995). Appearance of free radicals in the guinea pig inner ear after noise-induced acoustic trauma. Eur Arch Oto-Rhino-Laryngol.

[7] Yamashita D, Jiang HY, Schacht J, Miller JM (2004). Delayed production of free radicals following noise exposure. Brain Res.

[8] Henderson D, Bielefeld EC, Harris KC, Hu BH (2006). The role of oxidative stress in noise-induced hearing loss. Ear Hear.

[9] Robertson D (1983). Functional significance of dendritic swelling after loud sounds in the guinea pig cochlea. Hear Res.

[10] Abreu-Silva RS, Rincon D, Horimoto AR, Sguillar AP, Ricardo LA, Kimura L (2011). The search of a genetic basis for noise-induced hearing loss (NIHL). Ann Hum Biol.

[11] Park SN, Back SA, Park KH, Seo JH, Noh HI, Akil O (2013). Comparison of functional and morphologic characteristics of mice models of noise-induced hearing loss. Auris Nasus Larynx.

[12] Michael AK, Fribourgh JL, Chelliah Y, Sandate CR, Hura GL, Schneidman-Duhovny D (2017). Formation of a repressive complex in the mammalian circadian clock is mediated by the secondary pocket of CRY1. Proc Natl Acad Sci USA.

[13] Rosensweig C, Reynolds KA, Gao P, Laothamatas I, Shan Y, Ranganathan R (2018). An evolutionary hotspot defines functional differences between cryptochromes. Nat Commun.

[14] Fontana JM, Tserga E, Sarlus H, Canlon B, Cederroth C (2019). Impact of noise exposure on the circadian clock in the auditory system. J Acoust Soc Am.

[15] Takahashi JS (2017). Transcriptional architecture of the mammalian circadian clock. Nat Rev Genet.

[16] Basinou V, Park JS, Cederroth CR, Canlon B (2017). Circadian regulation of auditory function. Hear Res.

[17] Meltser I, Cederroth CR, Basinou V, Savelyev S, Lundkvist GS, Canlon B (2020). TrkB-mediated protection against circadian sensitivity to noise trauma in the murine cochlea. Curr Biol.

[18] Park JS, Cederroth CR (2016). Identification of a circadian clock in the inferior colliculus and its dysregulation by noise exposure. J Neurosci.

[19] Leake PA, Akil O, Lang H (2020). Neurotrophin gene therapy to promote survival of spiral ganglion neurons after deafness. Hear Res.

[20] Hernández-Echeagaray E (2020). Neurotrophin-3 modulates synaptic transmission. Vitam Horm.

[21] Fariñas I, Jones KR, Tessarollo L, Vigers AJ, Huang E, Kirstein M (2001). Spatial shaping of cochlear innervation by temporally regulated neurotrophin expression. J Neurosci.

[22] Fritzsch B, Pirvola U, Ylikoski J (1999). Making and breaking the innervation of the ear: neurotrophic support during ear development and its clinical implications. Cell Tissue Res.

[23] Rubel EW, Fritzsch B (2002). Auditory system development: primary auditory neurons and their targets. Annu Rev Neurosci.

[24] Yang T, Kersigo J, Jahan I, Pan N, Fritzsch B (2011). The molecular basis of making spiral ganglion neurons and connecting them to hair cells of the organ of Corti. Hear Res.

[25] Yang G, Yang Y, Tang H, Yang K (2020). Loss of the clock gene Per1 promotes oral squamous cell carcinoma progression via the AKT/mTOR pathway. Cancer Sci.

[26] Zheng Y, Liu C, Li Y, Jiang H, Yang P, Tang J (2019). Loss-of-function mutations with circadian rhythm regulator Per1/Per2 lead to premature ovarian insufficiency. Biol Reprod.

[27] Shafi AA, McNair CM, McCann JJ, Alshalalfa M (2021). The circadian cryptochrome, CRY1, is a pro-tumorigenic factor that rhythmically modulates DNA repair. Nat Commun.

[28] Lavebratt C, Sjöholm LK, Soronen P, Paunio T, Vawter MP, Bunney WE (2010). CRY2 is associated with depression. PLoS ONE.

[29] Jiang R, Duan J, Windemuth A, Stephens JC, Judson R, Xu C (2003). Genome-wide evaluation of the public SNP databases. Pharmacogenomics.

[30] Szobota S, Mathur PD, Siegel S, Black K, Saragovi HU, Foster AC (2019). BDNF, NT-3 and Trk receptor agonist monoclonal antibodies promote neuron survival, neurite extension, and synapse restoration in rat cochlea ex vivo models relevant for hidden hearing loss. PLoS ONE.

[31] Green SH, Bailey E, Wang Q, Davis RL (2012). The Trk A, B, C's of neurotrophins in the cochlea. Anat Rec.

[32] Fritzsch B, Tessarollo L, Coppola E, Reichardt LF (2004). Neurotrophins in the ear: their roles in sensory neuron survival and fiber guidance. Prog Brain Res.

[33] Chen S, Wang J, Cicek E, Roeder K, Yu H, Devlin B (2020). De novo missense variants disrupting protein–protein interactions affect risk for autism through gene co-expression and protein networks in neuronal cell types. Mol Autism.

[34] Chen S, Fragoza R, Klei L, Liu Y, Wang J, Roeder K (2018). An interactome perturbation framework prioritizes damaging missense mutations for developmental disorders. Nat Genet.

[35] Liu S, Liu L, Niu X, Lu D, Xia H, Yan S (2015). A novel missense mutation (I26M) in DUOXA2 causing congenital goiter hypothyroidism impairs NADPH oxidase activity but not protein expression. J Clin Endocrinol Metab.

[36] Hoste C, Rigutto S, Van Vliet G, Miot F, De Deken X (2010). Compound heterozygosity for a novel hemizygous missense mutation and a partial deletion affecting the catalytic core of the H2O2-generating enzyme DUOX2 associated with transient congenital hypothyroidism. Hum Mutat.

[37] Yonghong Zhou CD (2007). General biology.

[38] Miyagawa T, Hida A, Shimada M, Uehara C, Nishino Y, Kadotani H (2019). A missense variant in PER2 is associated with delayed sleep-wake phase disorder in a Japanese population. J Hum Genet.

[39] Chu LW, Zhu Y, Yu K, Zheng T, Yu H, Zhang Y (2008). Variants in circadian genes and prostate cancer risk: a population-based study in China. Prostate Cancer Prostatic Dis.

[40] Partch CL, Green CB, Takahashi JS (2014). Molecular architecture of the mammalian circadian clock. Trends Cell Biol.

[41] Menet JS, Abruzzi KC, Desrochers J, Rodriguez J, Rosbash M (2010). Dynamic PER repression mechanisms in the Drosophila circadian clock: from on-DNA to off-DNA. Genes Dev.

[42] Koike N, Yoo SH, Huang HC, Kumar V, Lee C, Kim TK (2012). Transcriptional architecture and chromatin landscape of the core circadian clock in mammals. Science.

[43] Ye R, Selby CP, Chiou YY, Ozkan-Dagliyan I, Gaddameedhi S, Sancar A (2014). Dual modes of CLOCK:BMAL1 inhibition mediated by cryptochrome and period proteins in the mammalian circadian clock. Genes Dev.

[44] Chiou YY, Yang Y, Rashid N, Ye R, Selby CP, Sancar A (2016). Mammalian Period represses and de-represses transcription by displacing CLOCK-BMAL1 from promoters in a Cryptochrome-dependent manner. Proc Natl Acad Sci USA.

[45] Yang CH, Hwang CF, Chuang JH, Lian WS, Wang FS, Huang EI (2020). Constant light dysregulates cochlear circadian clock and exacerbates noise-induced hearing loss. Int J Mol Sci.

[46] Yang CH, Hwang CF, Lin PM, Chuang JH, Hsu CM, Lin SF (2015). Sleep disturbance and altered expression of circadian clock genes in patients with sudden sensorineural hearing loss. Medicine.

[47] Mahmoudian-Sani MR, Mehri-Ghahfarrokhi A, Ahmadinejad F, Hashemzadeh-Chaleshtori M, Saidijam M, Jami MS (2017). MicroRNAs: effective elements in ear-related diseases and hearing loss. Eur Arch Otorhinolaryngol.

[48] Zhao L, Wang J, Li Y, Song T, Wu Y, Fang S (2021). NONCODEV6: an updated database dedicated to long non-coding RNA annotation in both animals and plants. Nucleic Acids Res.

[49] Chen J, Zhao X, Cui L, He G, Wang X, Wang F (2020). Genetic regulatory subnetworks and key regulating genes in rat hippocampus perturbed by prenatal malnutrition: implications for major brain disorders. Aging (Albany NY).

[50] Zhou X, Li Q, Xu J, Zhang X, Zhang H, Xiang Y (2016). The aberrantly expressed miR-193b-3p contributes to preeclampsia through regulating transforming growth factor-β signaling. Sci Rep.

[51] Jin G, Xu M, Zou M, Duan S (2020). The processing, gene regulation, biological functions, and clinical relevance of N4-acetylcytidine on RNA: a systematic review. Mol Ther Nucleic Acids.

[52] Arango D, Sturgill D, Alhusaini N, Dillman AA, Sweet TJ, Hanson G (2018). Acetylation of cytidine in mRNA promotes translation efficiency. Cell.

[53] Dominissini D, Rechavi G (2018). N(4)-acetylation of cytidine in mRNA by NAT10 regulates stability and translation. Cell.

[54] Zhang Q, Liu H, Soukup GA, He DZ (2014). Identifying microRNAs involved in aging of the lateral wall of the cochlear duct. PLoS ONE.

[55] Zheng S, Zhao T, Yuan S, Yang L, Ding J, Cui L (2019). Immunodeficiency promotes adaptive alterations of host gut microbiome: an observational metagenomic study in mice. Front Microbiol.

[56] Wu Y, Cao H, Baranova A, Huang H, Li S, Cai L (2020). Multi-trait analysis for genome-wide association study of five psychiatric disorders. Transl Psychiatry.

[57] Jiang L, Wang K, Lo K, Zhong Y, Yang A, Fang X (2019). Sex-Specific Association of circulating ferritin level and risk of type 2 diabetes: a dose-response meta-analysis of prospective studies. J Clin Endocrinol Metab.

[58] Wang X, Wu W, Zheng W, Fang X, Chen L, Rink L (2019). Zinc supplementation improves glycemic control for diabetes prevention and management: a systematic review and meta-analysis of randomized controlled trials. Am J Clin Nutr.

[59] Ji H, Dai D, Wang Y, Jiang D, Zhou X, Lin P (2015). Association of BDNF and BCHE with Alzheimer's disease: Meta-analysis based on 56 genetic case-control studies of 12,563 cases and 12,622 controls. Exp Ther Med.

[60] Xu M, Lin Z (2011). Genetic influences of dopamine transport gene on alcohol dependence: a pooled analysis of 13 studies with 2483 cases and 1753 controls. Prog Neuropsychopharmacol Biol Psychiatry.

[61] Yu H, Pan R, Qi Y, Zheng Z, Li J, Li H (2020). LEPR hypomethylation is significantly associated with gastric cancer in males. Exp Mol Pathol.

[62] Liu M, Li F, Yan H, Wang K, Ma Y, Shen L (2020). A multi-model deep convolutional neural network for automatic hippocampus segmentation and classification in Alzheimer's disease. Neuroimage.

[63] Wang X, Fang X, Zheng W, Zhou J, Song Z, Xu M, Min J, Wang F. Genetic support of a causal relationship between iron status and type 2 diabetes: a Mendelian randomization study. J Clin Endocrinol Metab. 2021:dgab454. 10.1210/clinem/dgab454. 10.1210/clinem/dgab454 PMC8530720 34147035

[64] Zhang F, Baranova A, Zhou C, Cao H, Chen J, Zhang X (2021). Causal influences of neuroticism on mental health and cardiovascular disease. Hum Genet.

[65] Zhang F, Rao S, Cao H, Zhang X, Wang Q, Xu Y, Sun J, Wang C, Chen J, Xu X, Zhang N, Tian L, Yuan J, Wang G, Cai L, Xu M, Baranova A. Genetic evidence suggests posttraumatic stress disorder as a subtype of major depressive disorder. J Clin Invest. 2021:145942. 10.1172/JCI145942. 10.1172/JCI145942 PMC8803333 33905376

[66] Hou L, Xu M, Yu Y, Sun X, Liu X, Liu L (2020). Exploring the causal pathway from ischemic stroke to atrial fibrillation: a network Mendelian randomization study. Mol Med.

