# Supplementary material for: A missense variant rs2585405 in clock gene PER1 is associated with the increased risk of noise-induced hearing loss in a Chinese occupational population
Source: BMC Med Genomics. 2021 Sep 8;14:221. doi: 10.1186/s12920-021-01075-x (PMC8425122; doi:10.1186/s12920-021-01075-x)
Supplement: Supplementary file 1 — Additional file 1 Table 6 Sensitivity analysis results of deleting age. The results of sensitivity analysis adjusted for sex, alcohol and tobacco use in a logistic regression model. [file 12920_2021_1075_MOESM1_ESM.docx]

Table 6 sensitivity analysis results of deleting age

| gene | Genetic models | Genotypes | Cases | | | Controls | | *P*^a^ | Adjusted OR  (95%CI)^b^ |
| --- | --- | --- | --- | --- | --- | --- | --- | --- | --- |
|  |  |  | n = 432 | | % | n = 837 | % |  |  |
| *PER1* | rs2585405 |  |  |  | |  |  |  |  |
|  | Codominant | GG | 98 | 22.7 | | 140 | 16.7 | **0.03** | 1.00 (Ref.) |
|  |  | GC | 198 | 45.8 | | 425 | 50.8 |  | 1.40 (1.01-1.95) |
|  |  | CC | 136 | 31.5 | | 272 | 32.5 |  | 0.93 (0.71-1.22) |
|  | Dominant | GG | 98 | 22.7 | | 140 | 16.7 | **0.01** | 1.00 (Ref.) |
|  |  | GC/CC | 334 | 77.3 | | 697 | 83.3 |  | 1.46 (1.10-1.95) |
|  | Recessive | GG/GC | 296 | 68.5 | | 565 | 67.5 | 0.71 | 1.00 (Ref.) |
|  |  | CC | 136 | 31.5 | | 272 | 32.5 |  | 1.05 (0.82-1.35) |
|  | Alleles | G | 394 | 31.0 | | 705 | 55.6 | 0.09 | 1.00 (Ref.) |
|  |  | C | 470 | 37.0 | | 969 | 76.4 |  | 1.15 (0.98-1.36) |
|  | rs2253820 |  |  |  | |  |  |  |  |
|  | Codominant | GG | 44 | 10.2 | | 96 | 11.5 | 0.45 | 1.00 (Ref.) |
|  |  | AG | 196 | 45.4 | | 399 | 47.7 |  | 0.82 (0.55-1.21) |
|  |  | AA | 192 | 44.4 | | 342 | 40.9 |  | 0.87 (0.68-1.12) |
|  | Dominant | GG | 44 | 10.2 | | 96 | 11.5 | 0.49 | 1.00 (Ref.) |
|  |  | AA/AG | 388 | 89.8 | | 741 | 88.5 |  | 0.88 (0.60-1.28) |
|  | Recessive | AG/GG | 240 | 55.6 | | 495 | 59.1 | 0.22 | 1.00 (Ref.) |
|  |  | AA | 192 | 44.4 | | 342 | 40.9 |  | 0.86 (0.68-1.09) |
|  | Alleles | G | 284 | 22.4 | | 591 | 46.6 | 0.22 | 1.00 (Ref.) |
|  |  | A | 580 | 45.7 | | 1083 | 85.3 |  | 0.89 (0.75-1.07) |
| *PER2* | rs56386336 |  |  |  | |  |  |  |  |
|  | Codominant | GG | 332 | 76.9 | | 661 | 79.0 | 0.50 | 1.00 (Ref.) |
|  |  | AG | 96 | 22.2 | | 165 | 19.7 |  | 1.35 (0.42-4.26) |
|  |  | AA | 4 | 0.9 | | 11 | 1.3 |  | 1.56 (0.48-5.03) |
|  | Dominant | GG | 332 | 76.9 | | 661 | 79.0 | 0.39 | 1.00 (Ref.) |
|  |  | AG/GG | 100 | 23.1 | | 176 | 21.0 |  | 0.88 (0.67-1.17) |
|  | Recessive | GG/AG | 428 | 99.1 | | 826 | 98.7 | 0.54 | 1.00 (Ref.) |
|  |  | AA | 4 | 0.9 | | 11 | 1.3 |  | 1.39 (0.44-4.39) |
|  | Alleles | G | 760 | 59.9 | | 1487 | 117.1 | 0.52 | 1.00 (Ref.) |
|  |  | A | 104 | 8.2 | | 187 | 14.7 |  | 1.09 (0.84-1.41) |
|  | rs934945 |  |  |  | |  |  |  |  |
|  | Codominant | GG | 242 | 56.0 | | 458 | 54.7 | 0.75 | 1.00 (Ref.) |
|  |  | AG | 157 | 36.3 | | 305 | 36.4 |  | 1.19 (0.76-1.84) |
|  |  | AA | 33 | 7.6 | | 74 | 8.8 |  | 1.16 (0.73-1.82) |
|  | Dominant | GG | 242 | 56.0 | | 458 | 54.7 | 0.66 | 1.00 (Ref.) |
|  |  | AG/AA | 190 | 44.0 | | 379 | 45.3 |  | 1.05 (0.83-1.33) |
|  | Recessive | AG/GG | 399 | 92.4 | | 763 | 91.2 | 0.47 | 1.00 (Ref.) |
|  |  | AA | 33 | 7.6 | | 74 | 8.8 |  | 1.17 (0.77-1.80) |
|  | Alleles | G | 641 | 50.5 | | 1221 | 96.2 | 0.50 | 1.00 (Ref.) |
|  |  | A | 223 | 17.6 | | 453 | 35.7 |  | 0.94 (0.79-1.13) |
| *CRY1* | rs1056560 |  |  |  | |  |  |  |  |
|  | Codominant | GG | 25 | 5.8 | | 54 | 6.5 | 0.37 | 1.00 (Ref.) |
|  |  | GT | 143 | 33.1 | | 306 | 36.6 |  | 0.85 (0.51-1.39) |
|  |  | TT | 264 | 61.1 | | 477 | 57.0 |  | 0.84 (0.66-1.08) |
|  | Dominant | GG | 25 | 5.8 | | 54 | 6.5 | 0.64 | 1.00 (Ref.) |
|  |  | GT/TT | 407 | 94.2 | | 783 | 93.5 |  | 0.90 (0.55-1.47) |
|  | Recessive | GT/GG | 168 | 38.9 | | 360 | 43.0 | 0.16 | 1.00 (Ref.) |
|  |  | TT | 264 | 61.1 | | 477 | 57.0 |  | 0.84 (0.67-1.07) |
|  | Alleles | G | 193 | 15.2 | | 414 | 326 | 0.18 | 1.00 (Ref.) |
|  |  | T | 671 | 52.9 | | 1260 | 99.3 |  | 0.88 (0.73-1.07) |
|  | rs3809236 |  |  |  | |  |  |  |  |
|  | Codominant | CC | 276 | 63.9 | | 514 | 61.4 | 0.59 | 1.00 (Ref.) |
|  |  | TC | 139 | 32.2 | | 282 | 33.7 |  | 1.28 (0.71-2.30) |
|  |  | TT | 17 | 3.9 | | 41 | 4.9 |  | 1.18 (0.65-2.15) |
|  | Dominant | CC | 276 | 63.9 | | 514 | 61.4 | 0.39 | 1.00 (Ref.) |
|  |  | TC/TT | 156 | 36.1 | | 323 | 38.6 |  | 1.11 (0.87-1.41) |
|  | Recessive | TC/CC | 415 | 96.1 | | 796 | 95.1 | 0.44 | 1.00 (Ref.) |
|  |  | TT | 17 | 3.9 | | 41 | 4.9 |  | 1.24 (0.70-2.22) |
|  | Alleles | C | 691 | 54.5 | | 1310 | 103.2 | 0.31 | 1.00 (Ref.) |
|  |  | T | 173 | 13.6 | | 364 | 28.7 |  | 0.90 (0.74-1.11) |
| *CRY2* | rs2292910 |  |  |  | |  |  |  |  |
|  | Codominant | CC | 36 | 8.3 | | 75 | 9.0 | 0.90 | 1.00 (Ref.) |
|  |  | AC | 180 | 41.7 | | 340 | 40.6 |  | 0.94 (0.61-1.44) |
|  |  | AA | 216 | 50.0 | | 422 | 50.4 |  | 1.04 (0.81-1.32) |
|  | Dominant | CC | 36 | 8.3 | | 75 | 9.0 | 0.71 | 1.00 (Ref.) |
|  |  | AC/AA | 396 | 91.7 | | 762 | 91.0 |  | 0.92 (0.61-1.40) |
|  | Recessive | AC/CC | 216 | 50.0 | | 415 | 49.6 | 0.89 | 1.00 (Ref.) |
|  |  | AA | 216 | 50.0 | | 422 | 50.4 |  | 1.02 (0.81-1.29) |
|  | Alleles | C | 252 | 19.9 | | 490 | 38.6 | 0.96 | 1.00 (Ref.) |
|  |  | A | 612 | 48.2 | | 1184 | 93.3 |  | 0.10 (0.83-1.19) |
|  | rs6798 |  |  |  | |  |  |  |  |
|  | Codominant | CC | 82 | 19.0 | | 173 | 20.7 | 0.60 | 1.00 (Ref.) |
|  |  | TC | 218 | 50.5 | | 398 | 47.6 |  | 0.96 (0.68-1.34) |
|  |  | TT | 132 | 30.6 | | 266 | 31.8 |  | 1.10 (0.84-1.43) |
|  | Dominant | CC | 82 | 19.0 | | 173 | 20.7 | 0.48 | 1.00 (Ref.) |
|  |  | TC/TT | 350 | 81.0 | | 664 | 79.3 |  | 0.90 (0.67-1.21) |
|  | Recessive | TC/CC | 300 | 69.4 | | 571 | 68.2 | 0.66 | 1.00 (Ref.) |
|  |  | TT | 132 | 30.6 | | 266 | 31.8 |  | 1.05 (0.82-1.36) |
|  | Alleles | C | 382 | 30.1 | | 744 | 58.6 | 0.91 | 1.00 (Ref.) |
|  |  | T | 482 | 38.0 | | 930 | 73.3 |  | 0.99 (0.84-1.17) |
| *BDNF* | rs11030099 |  |  |  | |  |  |  |  |
|  | Codominant | CC | 139 | 32.2 | | 250 | 29.9 | 0.65 | 1.00 (Ref.) |
|  |  | AC | 195 | 45.1 | | 398 | 47.6 |  | 1.07 (0.78-1.48) |
|  |  | AA | 98 | 22.7 | | 189 | 22.6 |  | 0.94 (0.70-1.27) |
|  | Dominant | CC | 139 | 32.2 | | 250 | 29.9 | 0.40 | 1.00 (Ref.) |
|  |  | AC/AA | 293 | 67.8 | | 587 | 70.1 |  | 1.11 (0.87-1.43) |
|  | Recessive | AC/CC | 334 | 77.3 | | 648 | 77.4 | 0.97 | 1.00 (Ref.) |
|  |  | AA | 98 | 22.7 | | 189 | 22.6 |  | 0.99 (0.75-1.31) |
|  | Alleles | C | 473 | 37.3 | | 898 | 70.8 | 0.60 | 1.00 (Ref.) |
|  |  | A | 391 | 30.8 | | 776 | 61.2 |  | 0.96 (0.82-1.13) |
|  | rs7124442 |  |  |  | |  |  |  |  |
|  | Codominant | CC | 1 | 0.2 | | 2 | 0.2 | 0.95 | 1.00 (Ref.) |
|  |  | TC | 58 | 13.4 | | 107 | 12.8 |  | 0.91(0.08-10.11) |
|  |  | TT | 373 | 86.3 | | 728 | 87.0 |  | 1.06(0.75-1.50) |
|  | Dominant | CC | 1 | 0.2 | | 2 | 0.2 | 1.00 | 1.00 (Ref.) |
|  |  | TC/TT | 431 | 99.8 | | 835 | 99.8 |  | 0.90(0.08-10.03) |
|  | Recessive | TC/CC | 59 | 13.7 | | 109 | 13.0 | 0.75 | 1.00 (Ref.) |
|  |  | TT | 373 | 86.3 | | 728 | 87.0 |  | 1.06(0.75-1.49) |
|  | Alleles | C | 60 | 4.7 | | 111 | 8.7 | 0.77 | 1.00 (Ref.) |
|  |  | T | 804 | 63.4 | | 1563 | 123.2 |  | 1.05(0.76-1.47) |
|  | rs6265 |  |  |  | |  |  |  |  |
|  | Codominant | GG | 125 | 28.9 | | 240 | 28.7 | 0.99 | 1.00 (Ref.) |
|  |  | AG | 204 | 47.2 | | 399 | 47.7 |  | 1.00 (0.72-1.38) |
|  |  | AA | 103 | 23.8 | | 198 | 23.7 |  | 0.98 (0.73-1.32) |
|  | Dominant | GG | 125 | 28.9 | | 240 | 28.7 | 0.92 | 1.00 (Ref.) |
|  |  | AA/AG | 307 | 71.1 | | 597 | 71.3 |  | 1.01 (0.78-1.31) |
|  | Recessive | AG/GG | 329 | 76.2 | | 639 | 76.3 | 0.94 | 1.00 (Ref.) |
|  |  | AA | 103 | 23.8 | | 198 | 23.7 |  | 1.01 (0.75-1.30) |
|  | Alleles | G | 454 | 35.8 | | 879 | 69.3 | 0.99 | 1.00 (Ref.) |
|  |  | A | 410 | 32.3 | | 795 | 62.6 |  | 1.00 (0.85-1.17) |
| *NTF3* | rs1805149 |  |  |  | |  |  |  |  |
|  | Codominant | AA | 97 | 22.5 | | 188 | 22.5 | 0.54 | 1.00 (Ref.) |
|  |  | AG | 67 | 15.5 | | 111 | 13.3 |  | 1.03 (0.77-1.37) |
|  |  | GG | 268 | 62.0 | | 538 | 64.3 |  | 1.21 (0.87-1.70) |
|  | Dominant | AA | 97 | 22.5 | | 188 | 22.5 | 1.00 | 1.00 (Ref.) |
|  |  | AG/GG | 335 | 77.5 | | 649 | 77.5 |  | 0.99 (0.75-1.31) |
|  | Recessive | AG/AA | 164 | 38.0 | | 299 | 35.7 | 0.43 | 1.00 (Ref.) |
|  |  | GG | 268 | 62.0 | | 538 | 64.3 |  | 1.10 (0.86-1.40) |
|  | Alleles | A | 261 | 20.6 | | 487 | 38.4 | 0.56 | 1.00 (Ref.) |
|  |  | G | 603 | 47.5 | | 1187 | 93.6 |  | 1.03 (0.90-1.18) |

^a^ Two-sided χ ^2^ test

^b^ Adjusted for sex, alcohol, and tobacco use in a logistic regression model
