# Supplementary material for: A missense variant rs2585405 in clock gene PER1 is associated with the increased risk of noise-induced hearing loss in a Chinese occupational population
Source: BMC Med Genomics. 2021 Sep 8;14:221. doi: 10.1186/s12920-021-01075-x (PMC8425122; doi:10.1186/s12920-021-01075-x)
Supplement: Supplementary file 4 — Additional file 4 Table 9 The results of sensitivity analysis in stratified analyses deleting tobacco. The results of sensitivity analysis in stratified analyses adjusted for age, sex and alcohol use in a logistic regression model. [file 12920_2021_1075_MOESM4_ESM.docx]

Table 9 The results of sensitivity analysis in stratified analyses deleting tobacco

| SNPs | Group | Genotype | Duration of noise exposed work (years) | | Expose level with noise (dB) | | |
| --- | --- | --- | --- | --- | --- | --- | --- |
|  |  |  | ≤16 | >16 | ≤85 | 85-92 | >92 |
| rs2585405 | case | GG | 27 | 71 | 63 | 26 | 9 |
|  |  | GC | 33 | 165 | 124 | 59 | 15 |
|  |  | CC | 24 | 112 | 78 | 41 | 17 |
|  | control | GG | 34 | 106 | 76 | 55 | 9 |
|  |  | GC | 80 | 345 | 248 | 139 | 38 |
|  |  | CC | 55 | 217 | 150 | 94 | 28 |
|  | p^a^ |  | 0.11 | 0.17 | **0.03** | 0.93 | 0.23 |
|  | Adjusted OR |  | 1.33 | 1.11 | 1.23 | 1.02 | 1.07 |
|  | (95%CI)^b^ |  | (0.93-1.91) | (0.92-1.34) | (0.99-1.53) | (0.76-1.38) | (0.62-1.84) |
|  | case | GG | 27 | 71 | 63 | 26 | 9 |
|  |  | GC/CC | 57 | 277 | 202 | 100 | 32 |
|  | control | GG | 34 | 106 | 76 | 55 | 9 |
|  |  | GC/CC | 135 | 562 | 398 | 233 | 66 |
|  | p^a^ |  | **0.04** | 0.07 | **0.01** | 0.72 | 0.16 |
|  | Adjusted OR |  | 1.86 | 1.37 | 1.64 | 1.08 | 1.85 |
|  | (95%CI)^b^ |  | (1.02-3.37) | (0.98-1.91) | (1.12-2.38) | (0.64-1.83) | (0.66-5.18) |

^a^ Two-sided χ ^2^ test

^b^ Adjusted for age, sex and alcohol use in a logistic regression model
